# Notes and News

**Published:** 1892-06-04

**Authors:** 


### Notes and News.

COLLECTED AND COMMUNICATED.

French charities have greatly benefited by the terms of Monsieur Finance's will. He has left 80,000 francs to be equally divided amongst twenty charity boards in the Paris districts, 70,000 francs to the like objects in the provinces, and 25,000 francs to the Orphanage at Vesinet; to the Ladies' Aid to the Wounded Association, 50,000 francs, the same sum to the Society for Assisting the Families of Shipwrecked Sailors, to the Villepint undertaking, the Night Refuge for Men, and the Night Refuge for Women.

At the ceremony held recently at the London Temperance Hospital, the Duchess of Westminster expressed great interest in the new children's ward, which she formally opened. The wards attracted many visitors. The casualty-room is conveniently placed near the entrance, and contains a most orderly array of "emergency" applications. The lavatories and bath-rooms attracted especial attention, being arranged on the most approved sanitary principles. The bath for the children's use is especially raised to a reasonable height, to suit both nurses and patients' convenience.

We can none of us shut our eyes to the fact that the narrower and crowded streets of London are sad places as the sole recreation grounds for the children of the very poor. Those who live in the vicinity of parks and open spaces are not the ones we plead for, but those little ones to whom even an occasional journey to such happy places, even falling far short as they do of real country resorts, are few and far between. In connection with *Pearson's Weekly* newspaper, there is a Fresh Air Fund, with which the secretary of the Ragged School Union co-operates. Through this medium those children to whom it will be, above all, beneficial, will be safely conveyed for holiday outings during the summer. The cost of these expeditions, including travelling and refreshment expenses is only 9d. a head, and surely there are few of our readers who would not be happier for the thought that they have made joyous the hearts of at least one or two poor children, at so small an outlay. The address of *Pearson's Weekly* is Temple Chambers, E.C., and we hope so useful a scheme will receive the assistance of our readers.

The Rose Show and Floral Fête to be held at the Mansion House on June 24th, in aid of the Royal Hospital for Women and Children, is a very novel and happy idea of the Lady Mayoress. Novelty, after all, is perhaps the most attractive attribute of any entertainment, and the Mansion House, under so unusual an aspect, is sure to draw the public should curiosity alone be the motive power. The Lady Mayoress has been fortunate in securing a large number of influential lady assistants, who cannot fail to secure the success of the fête.

Hospital Saturday at Birmingham has come and gone, and Birmingham may be justly proud that enthusiasm in a good cause shows no sign of flagging. When all the sums are paid into the bank it is anticipated that little short of £12,500 will be reached. Mr. Smedley and the Misses Stokes doubtless do more than can possibly be over-estimated in keeping the ball rolling. Such a memorial of an admirable scheme as the Convalescent Home was the happiest of ideas. Contributors feel they have an abiding testimony to the solid usefulness of the fund to which they are contributing, which stimulates all to laudable efforts in the good cause.

We are glad to learn that the bazaar, held in aid of the Great Northern Central Hospital, at Hampstead, was a great success, and that it will materially aid the hospital. The proceeds are to be devoted to the Ladies' Association Building Fund, which is making an effort to raise £5,000 for a women's ward in the new wing, to be erected in memory of the Duke of Clarence. In previous years the Ladies' Association have been very successful in their efforts. To them is due the out-patients' department, at the cost of £5,000, and we hope they will succeed in raising a like sum for the present purpose. The Duchesses of Portland, Westminster, and Rutland lent valuable assistance in severally opening the bazaar on the three days of its existence.

The Paris Board of Charities (*Assistance Publique*) is about to increase its charitable establishments to a large extent, utilising for this purpose seven and half million francs which remain from the loan granted in 1886. It is proposed that these institutions should include a hospital for chronic cases containing 400 beds at Brevanne to relieve the Paris hospitals. (2) A consumptive hospital at Blancheface for cases supposed to be curable. At present only one such establishment exists in Europe, that at Falkenstein, in Austria. (3) A hospital and dispensary for children suffering from acute or infectious diseases only, to be built on land belonging to the City of Paris, at the conjunction of three thoroughfares. (4) An infirmary for ringworm, together with an elementary school for day boarders at St. Louis. (5) The enlargement of the Rouchfoucauld Almshouses. (6) The reconstruction of the St. Antoine Isolation Hospital for Adults; and in connection with this a department for children, with pavilions for doubtful cases of measles and whooping-cough, and also an establishment for diphtheria convalescents outside Paris. (7) An immergency hospital in the Place du Danube, for influenza and other epidemics; and lastly, (8) two new maternity hospitals, one at Beaujon and the other at St. Antoine. If all these projects are carried into effect, the efficiency of the hospital service of Paris will be immensely augmented.

